# Initial Bonding Performance to CAD/CAM Restorative Materials: The Impact of Stepwise Concentration Variation in 8-Methacryloxyoctyl Trimethoxy Silane and 3-Methacryloxypropyl Trimethoxy Silane on Feldspathic Ceramic, Lithium Disilicate Glass-Ceramic, and Polymer-Infiltrated Ceramic

**DOI:** 10.3390/ma18091983

**Published:** 2025-04-27

**Authors:** Yukinori Maruo, Miho Kuwahara, Kumiko Yoshihara, Masao Irie, Noriyuki Nagaoka, Mai Yoshizane, Takuya Matsumoto, Kentaro Akiyama

**Affiliations:** 1 Department of Prosthodontics, Okayama University, 2-5-1 Shikata-cho, Okayama 700-8525, Japan; 2 Department of Occlusal and Oral Functional Rehabilitation, Okayama University Graduate School of Medicine, Dentistry and Pharmaceutical Sciences, 2-5-1 Shikata-cho, Kita-ku, Okayama 700-8525, Japan; kuwahara.miho@s.okayama-u.ac.jp (M.K.); yoshizane-m@s.okayama-u.ac.jp (M.Y.); akentaro@md.okayama-u.ac.jp (K.A.); 3 Health Research Institute, National Institute of Advanced Industrial Science and Technology, 2217-14 Hayashi-cho, Takamatsu 761-0395, Japan; kumiko.yoshihara@aist.go.jp; 4 Department of Biomaterials, Okayama University Graduate School of Medicine, Dentistry and Pharmaceutical Sciences, 2-5-1 Shikata-cho, Kita-ku, Okayama 700-8525, Japan; mirie@md.okayama-u.ac.jp (M.I.); tmatsu@md.okayama-u.ac.jp (T.M.); 5 Advanced Research Center for Oral and Craniofacial Sciences, Okayama University Dental School, 2-5-1 Shikata-cho, Kita-ku, Okayama 700-8525, Japan; nagaoka@okayama-u.ac.jp

**Keywords:** silane coupling, bond strength, ceramic, feldspathic, lithium, polymer-infiltrated ceramic, CAD/CAM

## Abstract

This study investigated the effects of varying concentrations of two distinct silane agents, 8-methacryloxyoctyl trimethoxy silane (8-MOTS) and 3-methacryloxypropyl trimethoxy silane (γ-MPTS), on their initial bonding efficacy to feldspathic ceramic (FC), lithium disilicate glass-ceramic (LD) and polymer-infiltrated ceramic (PIC) specimens, in 10% increments for concentrations ranging from 10% to 40%. Shear bond strengths between the ceramic substrates and the luting material were assessed following 24 h incubation in distilled water. For FC, the median value of shear bond strength peaked at 20% of γ-MPTS (7.4 MPa), while 8-MOTS exhibited a concentration-dependent increase, reaching its highest value at 40% (13.1 MPa). For LD, γ-MPTS above 10% yielded similar strength median values (10.2 MPa), whereas 8-MOTS at 30% (15.8 MPa) and 40% (13.4 MPa) yielded higher strength values than at 10% (2.9 MPa) and 20% (4.1 MPa), with the highest median value exhibited at 30%. For PIC, both γ-MPTS and 8-MOTS demonstrated similarly low bond strength values which were not significantly different from the non-silane-treated specimens. When applied on silica-based FC and LD, silane revealed a concentration-dependent bonding effect, with 8-MOTS exhibiting superior bond strength to γ-MPTS. However, PIC, characterized by a high inorganic filler content, demonstrated limited bondability with both silanes.

## 1. Introduction

Metal-free restorations, such as feldspathic ceramic, lithium disilicate glass-ceramic and polymer-infiltrated ceramic, have become preferred options in modern dentistry due to their ability to address esthetic concerns and circumvent metal-allergy sensitivities of patients [1,2,3]. These materials are also being used more frequently thanks to progress in digital technologies like intraoral scanning, computer-aided design (CAD), and computer-aided manufacturing (CAM). These technologies simplify the fabrication process, enhance precision by reducing material shrinkage and expansion, and lessen the reliance on extensive manual work [4,5,6].

Despite their esthetic superiority, biocompatibility and stable chemical properties, these materials typically exhibit lower mechanical strength compared with their metal counterparts [1,4,6]. To achieve optimal longevity for these restorations in the challenging oral environment, effective integration with the remaining tooth structure is especially crucial. Traditionally, such integration requires significant coronal taper reduction and surface conditioning [7].

Several factors affect the bond strength between a dental material and tooth substrate: surface treatment, adhesive system and cementation process. Studies have shown that bond strength can be enhanced by a combination of mechanical and chemical surface treatment strategies [8,9,10]. Mechanical surface treatment methods include airborne particle abrasion and hydrofluoric acid etching; chemical surface treatment is typically facilitated by functional monomers or silane coupling agents [8,9,10].

On mechanical treatment by air abrasion and acid etching, there are concerns that these methods induce surface crack propagation. Air abrasion, especially with alumina particles, removes portions of the polymer matrix and inorganic filler particles to create not only micro-retentive grooves but also surface defects that may compromise mechanical strength [11]. These surface modifications may lead to subsurface cracks and stress zones when under applied stress. Fracture occurs when stress intensity exceeds a threshold determined by a material’s composition and microstructure. Hence, the micro-retentive structure created by air abrasion significantly affects fracture toughness.

Chemical treatment has emerged as a highly effective method to establish durable ceramic-resin bonds, thus ensuring the treatment longevity of these esthetic materials [12]. Many commercially available surface treatment agents and luting materials incorporate functional adhesive monomers and silane coupling agents to elevate chemical interaction potential with materials, to the end of simplifying clinical procedures and reducing technique sensitivity [13,14].

In adhesive dentistry, silane coupling agents, notably 3-methacryloxypropyl trimethoxy silane (γ-MPTS), play a pivotal role by forming covalent bonds to promote adhesion at the interface between materials and luting agents through hydrolysis, condensation, hydrogen bonding and covalent bond formation [15]. However, in clinical practice, a wet oral environment presents challenges such as saliva contamination. Dental applications are hence subject to shorter processing times, lower temperatures and pressures compared with a controlled industrial environment. To improve adhesive strength under challenging silane treatment conditions, surface conditioning techniques such as heat treatment or vaporized adsorption of silane coupling agents have been proposed [16,17,18].

After hydrolysis, the methoxy groups thus created undergo condensation. Oligomers thus created from condensation form hydrogen bonds with -OH groups on the material substrate. Finally, covalent links are formed with the substrate through the condensation reaction. While a monolayer of silane-based primer is preferred, multilayer adsorption involving physical adsorption and chemisorption typically occurs [19,20]. Physical adsorption may result in thick layers prone to cohesive destruction between silane layers, posing a potential threat to bond strength. In contrast, chemisorption of thin monolayers, typically 10–50 nm thick, is more effective in achieving higher mechanical strength.

The hydrocarbon chain ((CH_2_)*n*) in silane coupling agents serves as a spacer between organo-functional terminal groups and hydrolysable groups, significantly impacting various monomer properties, including flexibility, solubility, wettability and the balance between hydrophobic and hydrophilic characteristics [21,22]. Longer hydrocarbon chains offer enhanced protection against self-condensation, leading to the formation of dimers, trimers and oligomers with greater solubility. Increased hydrophobicity facilitates compatibility between the monomer and polymer, enhances filler density and increases alkaline resistance in polymerized materials. Additionally, greater flexibility provided by longer carbon chains enhances the functional group’s reactivity and adhesion, ultimately resulting in greater flexibility of the polymerized material. The longer hydrocarbon chain of 8-MOTS exhibited enhanced hydrophobic behavior, demonstrating superior water repellency. Additionally, dental composites incorporating 8-MOTS exhibited significantly lower water sorption and solubility properties when compared to those with γ-MPTS [21,23].

This study examined and compared the chemical interaction potential of 8-methacryloxyoctyl trimethoxy silane (8-MOTS) with feldspathic ceramic (FC), lithium disilicate glass-ceramic (LD) and polymer-infiltrated ceramic (PIC) against that of γ-MPTS, noting the longer hydrocarbon chain of 8-MOTS. Additionally, we investigated and compared the effects of stepwise concentrations of these two silane agents on multilayer adsorption for bonding ability. The null hypothesis posited that the utilization of different silanes and their varying concentrations would not affect the initial bonding performance of CAD/CAM restorative materials comprising FC, LD and PIC.

## 2. Materials and Methods

Table 1 lists the CAD/CAM materials used in this study: FC (ARCTICA VITA Mark II, VITA Zahnfabrik, Bad Säckingen, Germany), LD (IPS e.max CAD, Ivoclar Vivadent AG, Schaan, Liechtenstein) and PIC (KATANA AVENCIA Block, Kuraray Noritake Dental, Tokyo, Japan).

Ninety specimens of each CAD/CAM material were prepared and randomly divided into nine groups (10 specimens per group) according to surface treatment method. Before surface treatment, all adhesive surfaces were polished with abrasive paper (Silicon Carbide abrasive paper, #2000, Struers A/S, Rodovre, Denmark) under water irrigation.

### 2.1. Surface Treatment

**Figure 1:**
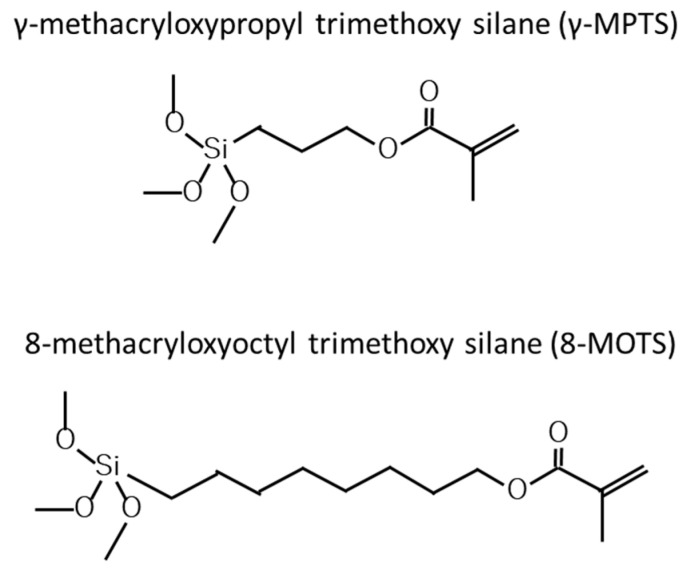
Structural formulas of silane coupling agents used in this study.

A polished surface was treated with a silane coupling agent, γ-MPTS or 8-MOTS (Figure 1), in four concentrations—10% to 40%—in 10% increments in ethanol for 30 s. Silane coupling solutions were activated using a hydrolysis solution of 2 wt% acetic acid (Table 1) just before use and applied to the adhesive surface with a commercially available disposable sponge brush for applying some primer and without any rinsing after application of the silane coupling agent.

Non-silane-treated specimens treated with acetic acid solution alone were prepared as controls.

### 2.2. Shear Bond Strength Test

**Figure 2:**
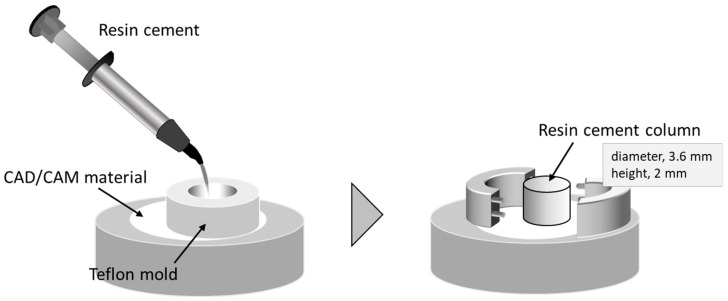
Schematic illustration of shear bond strength test. Resin cement column was built up on adhesive surface with a Teflon jig mold.

After a gentle blow-dry with oil-free air for 5 s after silane treatment, an adhesive resin cement (PANAVIA Veneer LC, Kuraray Noritake Dental, Tokyo, Japan; Table 1) was poured into a Teflon jig mold placed on the adhesive surface to form a resin cement column of 2 mm thickness and 3.6 mm diameter (Figure 2). Resin cement polymerization was performed with light-curing for 20 s with a visible light curing unit (New Light VL-II, GC, Tokyo, Japan).

**Figure 3:**
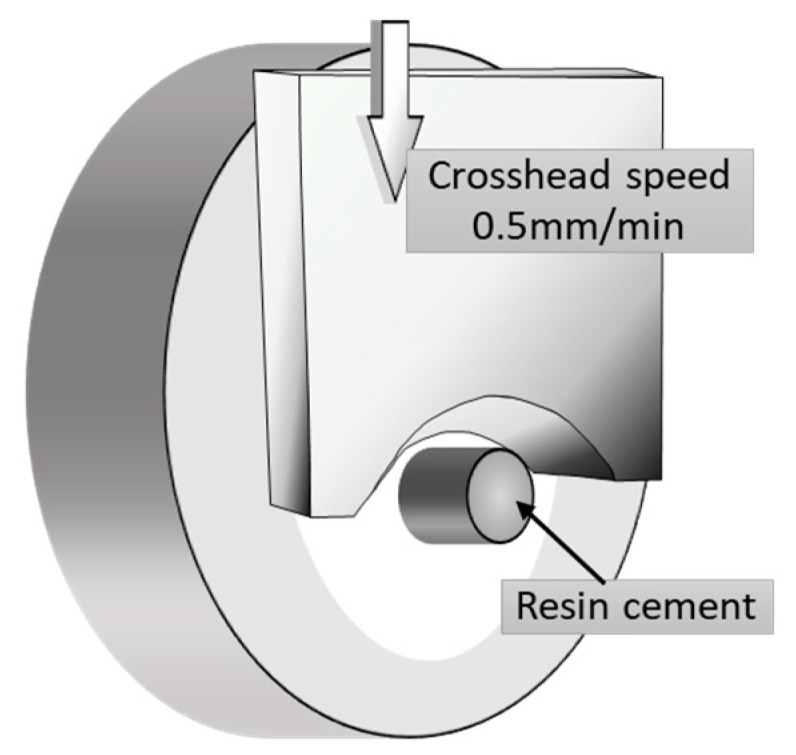
Shear bond strength was measured using a universal testing machine with a crosshead speed of 0.5 mm/min.

All bonded specimens were soaked in distilled water at 37 ± 2 °C for 24 h prior to the shear bond strength test. Each specimen was positioned in a shear test fixture, and the shear bond strength was determined using a universal testing machine (Autograph AG-X, Shimadzu, Kyoto, Japan) at a crosshead speed of 0.5 mm/min (Figure 3). The stress at failure was automatically calculated and recorded as the shear bond strength using specialized software.

### 2.3. Failure Analysis and Statistical Analysis

**Figure 4:**
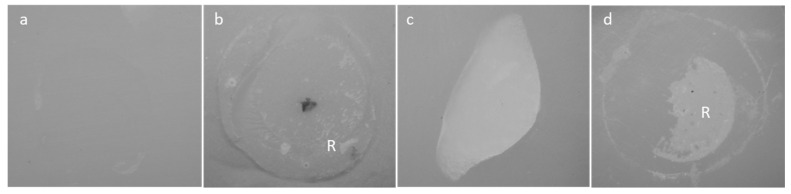
Exemplary pictures of samples after shear bond strength. (**a**) adhesive failure between resin cement and material; (**b**) cohesive failure within resin cement; (**c**) cohesive failure within material; (**d**) mixed-mode failure. R: resin cement.

After debonding, the fractured surfaces of ceramic specimens were examined under a light microscope (SZX16, Olympus, Tokyo, Japan). Failure caused by shear fracture was classified into one of the following three types: (1) adhesive failure between resin cement and material; (2) cohesive failure within resin cement; (3) cohesive failure within material, or (4) mixed-mode failure (adhesive–cohesive) (Figure 4).

Shear bond strength data of each group in each material were statistically compared using the nonparametric Kruskal–Wallis test (*p* < 0.05) and the Steel–Dwass multiple comparison test (*p* < 0.05), as the data lacked homogeneity of variance (*p* < 0.05) according to Levene’s test.

## 3. Results

### 3.1. FC Shear Bond Strength

**Figure 5:**
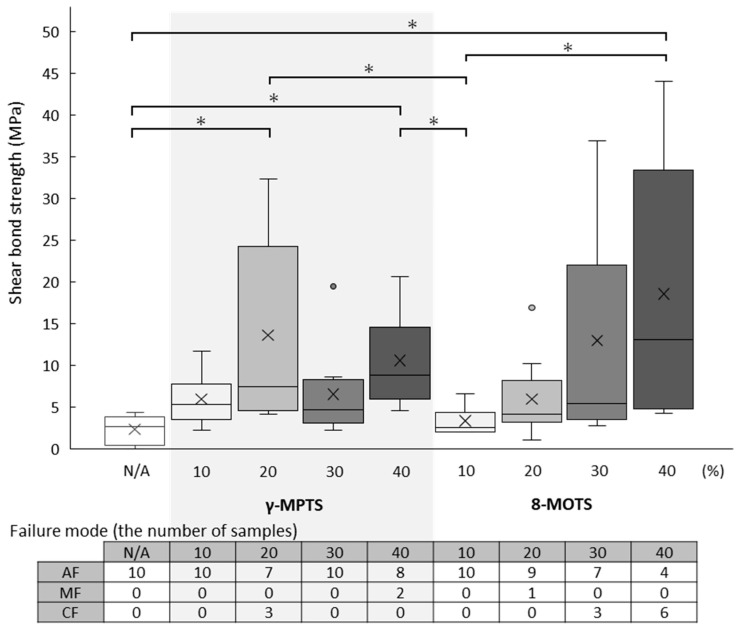
Shear bond strength values (MPa) of each group (upper) and failure mode (bottom) of FC. In the boxplot graph (upper), the horizontal line represents the median (50%), symbol “×” represents the mean and symbol “o” represents outlier. “*” indicates a significant difference in shear bond strength values between two groups (horizontal bar) based on the Kruskal–Wallis test (*p* < 0.05) and Steel–Dwass multiple comparison test (*p* < 0.05). In the table of failure mode, AF, MF and CF indicate adhesive failure, mixed failure and cohesive failure, respectively.

The shear bond strength values (median (minimum–maximum) MPa) and failure mode after the test of FC are shown in Table 2 and Figure 5.

With γ-MPTS, the highest shear bond strength value was detected at a 20% concentration (7.4 (4.2–32.4) MPa), which was significantly different from the untreated group (2.6 (0.0–4.3) MPa). Increasing concentration beyond 20% did not offer higher adhesive strength significantly different from that at 20%. All specimens showed adhesive failure except for three cohesive failures within FC at 20% and two mixed failures at 40%.

With 8-MOTS, there was a concentration-dependent increase in adhesive strength, reaching its highest value at 40% (13.1 (4.3–44.1) MPa). For cohesive failure within FC, three and six specimens were detected at 30% and 40%, respectively. For mixed failure, only one specimen was detected at 20%. The remaining specimens showed adhesive failure.

### 3.2. LD Shear Bond Strength

**Figure 6:**
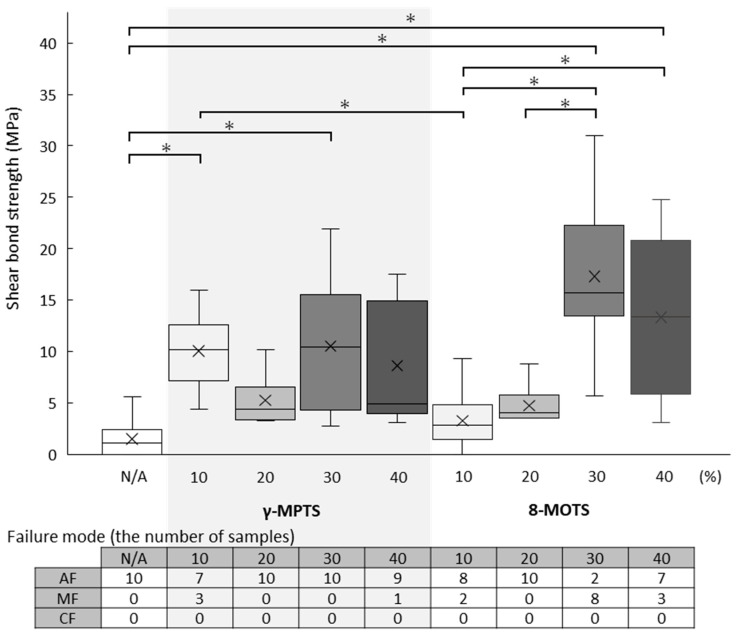
Shear bond strength values (MPa) of each group (upper) and failure mode (bottom) of LD. In the boxplot graph (upper), the horizontal line represents the median (50%) and symbol “×” represents the mean. “*” indicates a significant difference in shear bond strength values between two groups (horizontal bar) based on the Kruskal–Wallis test (*p* < 0.05) and Steel–Dwass multiple comparison test (*p* < 0.05). In the table of failure mode, AF, MF and CF indicate adhesive failure, mixed failure and cohesive failure, respectively.

The shear bond strength values (median (minimum–maximum) MPa) and failure mode after the test of LD are shown in Table 2 and Figure 6.

With γ-MPTS, almost the same adhesive strength value was yielded at all concentrations. When compared against the untreated group (1.1 (0.0–5.6) MPa), significant differences were detected at 10% (10.2 (4.4–16.0) MPa) and 30% (10.5 (2.8–21.9 MPa).

With 8-MOTS, adhesive strength values at concentrations of 30% (15.8 (5.7–31.0) MPa) were markedly increased when compared to 10% (2.9 (0.0–9.3) MPa) and 20% (4.1 (3.5–8.8) MPa), and at 40% (13.4 (3.1–24.8) MPa), adhesive strength values were markedly increased when compared to 10%. The 30% concentration yielded the highest adhesive strength value, significantly different from those at 10% and 20%.

### 3.3. PIC Shear Bond Strength

**Figure 7:**
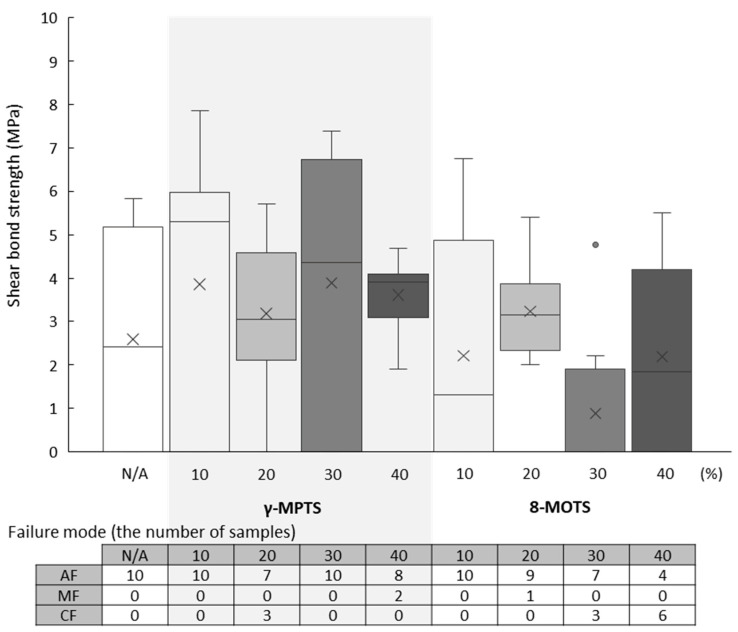
Shear bond strength values (MPa) of each group (upper) and failure mode (bottom) of PIC. In the boxplot graph (upper), the horizontal line represents the median (50%), symbol “×” represents the mean and symbol “o” represents outlier. There is not a significant difference in shear bond strength values based on the Kruskal–Wallis test (*p* < 0.05) and Steel–Dwass multiple comparison test (*p* < 0.05). In the table of failure mode, AF, MF and CF indicate adhesive failure, mixed failure and cohesive failure, respectively.

The shear bond strength values (median (minimum–maximum) MPa) and failure mode after the test of PIC are shown in Table 2 and Figure 7.

Regardless of concentration, both γ-MPTS and 8-MOTS demonstrated similarly low bond strength values, which were not significantly different from the untreated group (2.4 (0.0–5.8) MPa). Adhesive failure or mixed failure was observed across all specimens except cohesive failure within PIC at 20% of γ-MPTS (3.1 (0–5.7) MPa) and at 30% (0 (0–4.8) MPa) and 40% (1.8 (0–5.5) MPa) of 8-MOTS.

## 4. Discussion

In dentistry, adhesion between a restoration and tooth structure is of paramount importance to a treatment’s longevity. To the end of integrating the restoration and tooth structure, myriad luting materials have been introduced. Luting agents and functional monomers are mainly composed of organic compounds; silane coupling agents and inorganic ingredients are commonly used to enhance the mechanical properties of dental materials and thus improve bonding.

Surface treatment with silane coupling agents for silica-based materials has been the most common pretreatment strategy for many years. Besides silica-based ceramics, silane coupling agents have increasingly been applied to esthetic materials used in digital dentistry, such as LD, PIC and microfilled resin.

Inorganic materials can be chemically bonded to organic materials of luting materials through pretreatment primers, such as the modification of inorganic surfaces with silane coupling agents. However, the priming procedure of silane coupling agents in the dental field is completely different from that used in the industrial world, where the latter allows for high temperatures and long treatment periods [15].

In dentistry, silane coupling agent primers are commercially available either in two bottles, with the silane coupling solution in one bottle and acetic acid or acidic adhesive monomer solution for activation in another bottle, or in a single bottle, within which these ingredients are already mixed in an acidic solution. During clinical practice, priming and luting are performed after trial fitting and occlusal adjustment of the restoration. The chairside priming procedure for inorganic restorative surface mandates a very short treatment time of only 30 s to several minutes under room temperature—be it single-bottle or two-bottle priming. To improve the reactivity of silane coupling agents within such a short time and at a lower temperature, other procedures such as deposition with a vaporized silane coupling agent or heat treatment after primer application have been advocated [24,25,26].

To obtain long-term stability, air abrasion or hydrofluoric acid etching are used in addition to priming to provide mechanical bonding force. Ironically, the mechanical properties of the restorative itself might be degraded, especially at the thin margin edges of the restoration, by air abrasion by virtue of its pressure, direction and duration [27,28]. These shortcomings of mechanical retention mechanisms bring the importance of chemical bonding with silane coupling agents into sharper focus. Since the focus of this study was to clarify the effects of chemical bonding with silane coupling agents, mirror-polished surfaces were used to preclude the influence of mechanical bonding.

After pretreatment with a silane coupling agent, both chemical and physical modes of adsorption exist on the material’s surface. Chemical bonding, not physical adsorption, is more effective for the long-term stability of the material luted with dental cement [27]. Therefore, the silane coupling agent should be of low concentration and applied in a small amount as a thin layer on the material—and this is indeed practiced on single standardized material surfaces in the industrial field. In the dental field, various materials’ surfaces are roughly treated chairside with a paint brush. Hence, unlike in industrial practice, conditions such as primer amount, treatment time and temperature cannot be specific and precise.

On the other hand, physical adsorption of a silane coupling agent would be effective to some degree at a higher concentration, since the silane coupling agent could penetrate the material and form a reticular structure. The concentration of silane coupling agents commercially available for dental materials is approximately 1–10%, and some agents are available at higher concentrations of 40–60% [15].

The most commonly used silane coupling agent is γ-MPTS, which has three hydrocarbon chains between the silanol group and the polymerizable group. Under wet conditions in the oral cavity, a silane coupling agent with a longer hydrocarbon chain offers higher hydrophobicity and stability against degradation when compared to γ-MPTS. After bonding, a long-hydrocarbon-chain silane coupling agent also confers greater flexibility [15]. For all these benefits, long-hydrocarbon-chain silane coupling agents have been incorporated into some commercially available luting materials and are also used to modify the surface of inorganic fillers in dental materials. However, the influence of longer-hydrocarbon-chain silane coupling agents for adhesion remains unclear at this stage.

In this study, silane pretreatment showed more bonding efficacy on FC than LD and PIC. Unlike the compositions of LD and PIC, silicon dioxide is the main ingredient in FC. Hence, there were more OH groups on the FC surface for bonding to silanol groups than on LD and PIC.

With FC, the adhesive strength of γ-MPTS peaked at 20% in this study, but it did not increase with increasing concentrations. Unlike γ-MPTS, the adhesive strength of 8-MOTS increased in a concentration-dependent manner. This difference between γ-MPTS and 8-MOTS could be attributed to improved flexibility after bonding with long-hydrocarbon-chain silane coupling agent or due to differences in the polarity and three-dimensional structure of the silane coupling ligands.

With LD, bond strength values were not as high as those of FC. The adhesive strength of γ-MPTS was not affected by concentration. For 8-MOTS, a pronounced increase to a higher bond strength occurred between 20% and 30% concentrations, indicating that a higher concentration could yield a higher bond strength. 8-MOTS had a longer hydrocarbon chain than γ-MPTS, and this property could have resulted itn a more complex structure entailing both chemisorption and physical absorption on the surface pretreated with 8-MOTS. However, physical adsorption is seemingly inferior to chemical adsorption for long-term stability; hence, the positive effects of 8-MOTS on LD remained unclear. Moreover, alcohol washing should be used after treatment to remove the deposited agent, including physical adsorption, on the material surface. This would ensure that silane coupling agent existed in a monolayer on the material surface to the end of obtaining the highest adhesive strength.

With PIC, the effect of the silane coupling agent on bonding was very low, showing almost the same low bond strength value regardless of concentration for both silane coupling agents. Hybrid resins consist of inorganic filler/structure and a resin matrix. While silane coupling agents may bond to the exposed inorganic component on the bonding surface, the abundance of zirconia fillers could have hindered the bonding of the incorporated inorganic component with the silane coupling agent, resulting in weak bond strengths obtained in this study. Moreover, the polymerization rate of the resin matrix was quite high because polymerization was carried out under high-temperature and high-pressure conditions during production. Consequently, the lack of unpolymerized monomers on the PIC surface resulted in inadequate bonding.

In this study, the silanized surfaces were not rinsed to remove physically absorbed, non-reacted silane molecules. At higher silane concentrations, physical adsorption may occur alongside chemical bonding. The purpose of using high concentrations of the silane coupling agent was to investigate both physical and chemical bonding effects. This was necessary because achieving monolayer deposition is extremely challenging under conditions such as the lower temperature and pressure during silane treatment, as well as the potential contamination from saliva. Currently, some commercial silanes used in dentistry have a high concentration of effective silane content of 40–60% γ-MPTS [15]. A silane monolayer typically ranges from 10 to 50 nm in thickness. While the term ‘monolayer deposition’ is commonly associated with the silanization grafting process, it is highly context-dependent. Excessive deposition of successive or thicker silane layers can lead to cohesive failure between the layers of physical and chemical bonding, ultimately weakening the mechanical integrity of the structure [23]. An ultrathin silane layer may result in weak mechanical bonding, making it ineffective in improving interfacial mechanical properties. Therefore, to achieve optimal mechanical performance, it is important to use an ideal silane coating thickness.

Long-term stability is a very important clinical factor. To obtain meaningful clinical feedback on long-term stability and durability, it is necessary to set conditions and interpret the data and results in the correct context. For example, a crucial problem in adhesive dentistry is the degradation of materials themselves, especially resin cements and ceramic materials [29]. Although durability tests like thermal cycling and long-term storage can certainly lead to a decrease in adhesive strength, the key question is whether this decline is due to material degradation or the loss of adhesive effectiveness. Nevertheless, the findings from these durability tests align with clinical observations, which show a synergistic reduction in both adhesive strength and the ceramic material itself. A limitation of this study is that it only assessed the initial bond strength without incorporating thermal cycling. Further investigation is required to address this gap.

From the results of this study alone, it is difficult to draw conclusions about which silane coupling agent is clinically effective for bonding to CAD/CAM restorative materials. Although the exact cause remains unclear, a limitation of this study is that the 10 samples per group could not be analyzed using more powerful parametric tests than the Kruskal–Wallis test and the Steel–Dwass multiple comparison test due to failure to meet the assumptions of the homogeneity of variance (*p* < 0.05) when subjected to Levene’s test. Nonetheless, initial results in this study showed that long-hydrocarbon-chain silane coupling agents could yield higher bond strengths at higher concentrations. On the other hand, short-hydrocarbon-chain silane coupling agents also seemed to be more effective than longer ones, although this remained unclear within the limitations of this study.

## 5. Conclusions

The effect of long-hydrocarbon-chain silane coupling agents was affected by inorganic components, including Si, on the material surface; however, a limitation of this study is that long-term stability was not studied. The initial bonding effects of silane on silica-based FC and LD revealed a concentration-dependent relationship, with 8-MOTS exhibiting superior bond strength when compared with γ-MPTS. However, PIC, characterized by high inorganic filler content, demonstrated limited bondability with both silanes.

## Figures and Tables

**Table 1:** Materials used in the study.

Brand Name	Description	Manufacturer
ARCTICA VITA Mark II (FC)	Feldspathic ceramic SiO_2_ (56–64%), Al_2_O_3_ (20–23%), Na_2_O (6–9%) and K_2_O (6–8%)	Vita Zahnfabrik Bad Säckingen, Germany
Litium discilicate ceramic (LD)	Lithium disilicate glass-ceramic SiO2 (57–80%), Li_2_O (11–19%), K_2_O (0–13%), P_2_O_2_ (0–11%), ZrO_2_ (0–8%), ZnO (0–8%), other oxides and ceramic pigments (0–10%)	Ivoclar Vivadent AG, Schaan, Liechtenstein
KATANA AVENCIA Block (PIC)	Polymer-infiltrated ceramic Al_2_O_3_ (20 nm) and SiO_2_ (40 nm) (62%), UDMA and TEGDMA	Kuraray Noritake Dental, Tokyo, Japan
Silane solution	γ-MPTS (10.0, 20.0, 30.0, 40.0 wt%) in ethanol 8-MOTS (10.0, 20.0, 30.0, 40.0 wt%) in ethanol	Shin-Etsu Chemical, Tokyo, Japan
Acetic acid solution	Acetic acid (2.0%), ethanol (70.0%) and water (28.0%)	Wako Pure Chemical Industries, Osaka, Japan
PANAVIA Veneer LC	Silanated spherical silica filler, UDMA, ytterbium trifluoride, TEGDMA, hydrophilic aliphatic dimethacrylate, hydrophilic amide monomer, accelerators, DL-Camphorquinone and pigments	Kuraray Noritake Dental, Tokyo, Japan

γ-MPTS: γ-methacryloxypropyl trimethoxy silane, 8-MOTS: 8-methacryloxyoctyl trimethoxy silane, UDMA: urethane dimethacrylate, TEGDMA: triethyleneglycol dimethacrylate.

**Table 2:** Shear bond strength value (median (minimum–maximum) MPa).

N/A	γ-MPTS				8-MOTS			
	10	20	30	40	10	20	30	40
2.6	5.3	7.4	4.7	8.8	2.6	4.1	5.4	13.1
(0–4.3)	(2.2–11.7)	(4.2–32.4)	(2.2–19.5)	(4.6–20.6)	(2.0–6.6)	(1.1–16.9)	(2.8–36.9)	(4.3–44.1)
1.1	10.2	4.4	10.5	5.0	2.9	4.1	15.8	13.4
(0–5.6)	(4.4–16.0)	(3.3–10.2)	(2.8–21.9)	(3.1–17.5)	(0–9.3)	(3.5–8.8)	(5.7–31.0)	(3.1–24.8)
2.4	5.3	3.1	4.4	3.9	1.3	3.2	0	1.8
(0–5.8)	(0–7.9)	(0–5.7)	(0–7.4)	(1.9–4.7)	(0–6.8)	(2.0–5.4)	(0–4.8)	(0–5.5)

n = 10 for each group. Each horizontal bar is indicated a significant difference (*p* < 0.05) within each material according to the Kruskal–Wallis test and Steel–Dwass multiple comparison test.

## Data Availability

The original contributions presented in the study are included in the article, further inquiries can be directed to the corresponding author.
